# Estimated number of children affected by paternal cancer diagnosis and death in Finland during 1970–2022: a population-based registry study

**DOI:** 10.2340/1651-226X.2026.45653

**Published:** 2026-07-30

**Authors:** Anniina Kyrönlahti, Eetu Mäkinen, Karri Seppä, Tea Lallukka, Janne Pitkäniemi

**Affiliations:** a Cancer Society of Finland, Finnish Cancer Registry, Helsinki, Finland; b Children and Adolescents, Helsinki University Hospital and University of Helsinki, Helsinki, Finland; c Faculty of Social Sciences, Tampere University, Tampere, Finland; d Department of Public Health, Faculty of Medicine, University of Helsinki, Helsinki, Finland

**Keywords:** Child, fathers, cancer, orphans, estimation, trend

## Abstract

**Background and purpose:**

Parental cancer effects millions of children worldwide. We estimated the number of children influenced by paternal cancer and paternal orphans, and examined changes in their numbers and annual rates over time, with a focus on time trends and the influence of cancer type.

**Patients and methods:**

Male cancer patients aged ≥15 years, diagnosed since 1953, were identified from the Finnish Cancer Registry. Cancer deaths and information on live births were obtained from Statistics Finland. Annual number of new children and prevalent children whose father was diagnosed with cancer and the annual number of new orphans and prevalent orphans due to paternal cancer death for 1970–2022 were estimated indirectly by combining cancer incidence and mortality data with population-level fertility rates.

**Results:**

The estimated age-standardized rate of new children with paternal cancer increased from 96/100,000 children in 1970–1974 to 163/100,000 in 2017–2021. The annual rate of new paternal orphans decreased from 56/100,000 in 1970–1974 to 35/100,000 in 2017–2021. The rate of new children increased 1.1% per year while the rate of new orphans decreased 1.1% in 1970–2021.

**Interpretation:**

Although the number of paternal orphans is decreasing due to improved cancer survival, the number of children influenced by paternal cancer is rising reflecting the increasing cancer incidence and resulting in more children vulnerable to the harmful effects of paternal cancer.

## Graphical abstract

**Figure:**
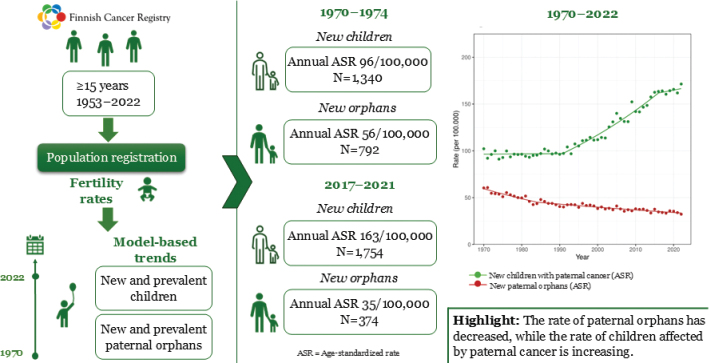

## Introduction

Globally, an estimated 10.3 million men of all ages are diagnosed with cancer each year, and 5.4 million men die from the disease [1]. In 2022, the cumulative risk of developing cancer before the age of 75 years was 21.8%, and the risk of dying from cancer before the age of 75 was 11.4% among males. Cancer impacts not only the cancer patients themselves but also the whole family. Global estimates of prevalent maternal orphans due to cancer were 7 million children in mid-2020s [2], but little is known about the number of paternal orphans. The annual estimates of minors effected by parental cancer have varied between 0.28% and 0.38% in population-based registry studies [3–5].

Few register studies have estimated the number of children affected solely by paternal cancer. In the 1987 Finnish Birth Cohort study, 1.8% of the children aged 0–17 years had a father with cancer over the 21-year follow-up period [6]. In a Danish population-based study, 2.1% of the children had experienced paternal cancer during 1986–1999 [7]. A Swedish register-based study showed that between 1961 and 2023, 3.4% (*n* = 126,696) of all children were born to men who had cancer at any point in their lives [8].

In Finland, the most common cancer type among men aged 20–69 years in 2022 was prostate cancer with the incidence of 125.8 per 100,000 person-years followed by colorectal cancer, lung and tracheal cancer, melanoma of the skin, and mature B-cell neoplasms [9]. Among adolescent males aged 15–19 years, lymphomas are the most common cancer type [10]. Cancer incidence among Finnish men has steadily increased up to 2003 reaching a plateau in the recent two decades [9].

In 2022, diseases of the circulatory system were the most common cause of death among males aged 15–64 years in Finland (*n* = 1,259), followed by neoplasms (*n* = 1,101), accidents and violence (*n* = 964), alcohol-related diseases and alcohol poisoning (*n* = 710), and suicides (*n* = 417) [11]. The most common cause of cancer death among men aged 20 to 69 years in 2022 was lung and tracheal cancer (mortality 25.6 per 100,000 person-years) followed by colorectal cancer and pancreatic cancer. Cancer mortality in men has been decreasing by 1.2% per year during the period of 2008–2022 [9].

The age of having children has gradually risen in the last decades and in 2023 the average age of becoming a father in Finland was 32.2 years [12]. As cancer risk increases with age alongside growing cancer prevalence [9], more children are expected to be affected by paternal cancer. At the same time, fathers’ caregiving roles have expanded, with increasing involvement in childcare and family leave [13]. Earlier studies have shown children and adolescents of cancer patients to be at higher risk for psychosocial problems [14] suggesting even more pronounced risk with paternal cancer [15]. The aforementioned details highlight the importance of knowing the amount of children impacted by paternal cancer and the possible consequences of the father’s cancer diagnosis on their children.

This is the first study to use longitudinal, comprehensive cancer registry data alongside reliable, registry-based paternal fertility rates to estimate age-standardized rates of children whose father was diagnosed with cancer and of children whose father died of cancer (paternal orphans). As an additional novel aspect, we also explored time trends in the rates of children newly affected by paternal cancer diagnosis (new children) and of children whose father had ever been diagnosed with cancer (prevalent children). In addition, we assessed both incident and prevalent paternal orphans resulting from cancer. Furthermore, we studied the influence of cancer type on the estimated number of children and orphans, as well as their trends over time.

## Patients/material and methods

All male cancer patients aged 15 years or older, diagnosed between 1953 and 2022, were identified from the Finnish Cancer Registry database [10, 16]. Causes of death (cancer deaths) were obtained from Statistics Finland [11].

Live births by the age of the father were available annually between 1990 and 2022 from Statistics Finland’s database [17], and fertility rates were calculated based on this information. In addition, total births between 1953 and 1989 were observed. Using an age-period-cohort Poisson model, number of births by father’s age between 1953 and 1989 were estimated and scaled so that the yearly sums of births by the father’s age equaled the observed yearly total number of births. Thus, this study did not use individual-level linkage between fathers and cancer cases. Instead, the number and rates of children were estimated indirectly by combining cancer incidence and mortality data with population-level fertility rates.

Cancer types were divided into their own categories based on ICD-10 coding system [18]. Using the cut-off limit of approximate proportion of 1% in incidence and mortality, cancer types below the limit were categorized as ‘Others’. The cancer grouping is presented in Table S1.

Based on the number of male cancer patients and cancer-related deaths, as well as estimated fertility rates, numbers and rates of underaged children affected by paternal cancer and paternal orphanhoods were estimated for the period 1970–2022. An underage child was defined as a person under the age of 18 and paternal orphans were children whose father had died before the child reached 18 years of age.

Annual number of children whose father was newly diagnosed with cancer (later referred as new children) and the number of orphans due to newly occurred paternal cancer death (later referred as new orphans) were estimated for the whole period 1970–2022. Total number of children (prevalent children) whose father was ever diagnosed with cancer and total number of paternal orphans (prevalent orphans) were also estimated for the whole period. Our approach means that children whose father has been diagnosed with cancer are included in the number of prevalent children with paternal cancer until the age of 18, regardless of the father’s death or recovery from cancer. Estimates were performed following the methodology introduced by Guida et al. in 2022 (Supplement: Statistical Methods) [2]. The age-standardized rates of children with paternal cancer and paternal orphans were estimated using the World (WHO 2000–2025) Standard.

Poisson regression was used to estimate the changepoints and piecewise trends (annual percentage change, APC) in annual new and total number of children influenced by paternal cancer and paternal orphans. APC was also estimated for the rate of new children with paternal cancer and new paternal orphans, and an additional analysis was made adjusting for the father’s age. Bayesian Information Criterion (BIC) was used to select the number of changepoints for each time series [19]. Variations in trends of new children influenced by paternal cancer diagnosis and new paternal orphans due to cancer death over time are demonstrated in Table 1 for 5-year calendar periods 1970–1974, and 2017–2021, and around the estimated changepoints. Estimates for year 2022 are reported separately. Analyses were performed in the programme R (version 4.3.2). All analyses were stratified by cancer type.

**Table 1:** Estimated numbers and the corresponding age-standardized rates (per 100.000) of children under 18 years of age influenced by paternal cancer diagnosis (at the age of ≥15 years) and paternal cancer death in Finland during 1970–2022 by the latest study year 2022, 5-year periods at the start and end of follow-up, and around estimated changepoints.

Population quantity		First period 1970–1974	First changepoint		Second changepoint		Last period 2017–2021	Year 2022
		Average value	Year	Average value	Year	Average value	Average value	Value
New children with paternal cancer	Number of children	1,340	1987	1,127	2016	1,741	1,754	1,832
	Age-standardized rate	95.8	1991	98.6	2016	161.4	163.1	171.1
Prevalent children with paternal cancer	Number of children	9,228	1987	7,581	2014	11,067	12,208	12,704
	Age-standardized prevalence	644.4	1989	673.4	2001	784.5	1115.9	1151.2
New orphans	Number of orphans	792	1990	482	1993	486	374	351
	Age-standardized rate	56.4	1984	44.9	-	-	34.5	32.3
Prevalent orphans	Number of orphans	5,680	1988	3,479	1998	3,338	2,580	2,530
	Age-standardized prevalence	395.6	1990	297.5	-	-	235.5	227.8

## Results

A total of 18,457 new cancers (age-standardized rate 442/100,000) among men aged 15 years or older were diagnosed in 2022, of which 2.9% occurred among men aged 15–39 years. Our study showed an increase in both cancer incidence and age at diagnosis; on the average 6,049 new cancers (388/100,000) were diagnosed annually between 1970 and 1974, of which 4.4% occurred among men aged 15–39 years. The estimated median age of the father at cancer diagnosis increased from 44.9 years in 1970–1974 to 47.3 years in 2017–2021.

In 2022, 6,968 new cancer deaths (147/100,000) occurred among men aged 15 years or older. Increase in the number of new cancer deaths over time was seen with the average number of 4,434 cancer deaths occurring between 1970 and 1974. The median age of father at cancer death increased from 45.4 years in 1970–1974 to 50.3 years in 2017–2021.

The estimated median age of children at paternal cancer diagnosis in 2017–2021 was 11.7 years, representing a slight decrease from 12.5 years in 1970–1974 (Table 1). The median age of children at paternal cancer death in 2017–2021 was 12.5 years, with no major changes over time. The lowest median age at both paternal cancer diagnosis and death in 2017–2021 was observed among children whose father had testicular cancer (diagnosis 7.0 years; death 7.5 years), whereas the highest median age was observed in association with paternal prostate cancer (diagnosis 13.9 years; death 13.9 years) (Table S2).

### Children influenced by paternal cancer diagnosis

**Figure 1:**
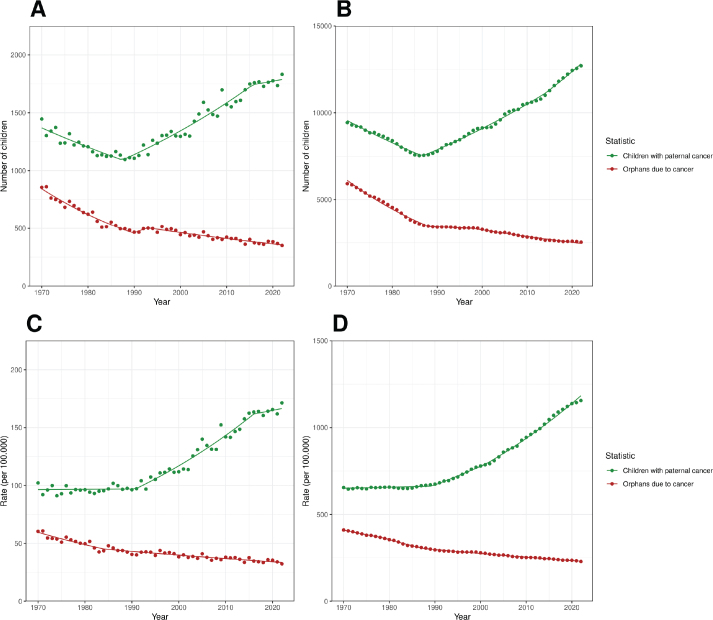
Estimated annual percentage changes (APCs) in the number of new children/orphans (A), in the number of prevalent children/orphans (B), in the age-standardized rate of new children/orphans (C), and in the age-standardized rate of prevalent children/orphans (D) under 18 years of age influenced by paternal cancer diagnosis (at the age of ≥15 years) and paternal cancer death (orphans) in Finland during 1970–2022.

In 2022, the estimated age-standardized rate of children newly influenced by paternal cancer diagnosis (new children) was 171 per 100,000 (Table 1). The average age-standardized rate of new children whose father was diagnosed with cancer has increased overall since the first study period of 1970–1974 from 96 per 100,000 to 167 per 100,000 in 2017–2021. In 1970– 2022, the average age-standardized rate of new children whose fathers were diagnosed with cancer increased by 1.1% per year (Table 2, Figure 1c). When adjusted for father’s age, the increase was attenuated (0.7% per year) (Table 2). Changepoints in the age-standardized rate in 1970–2022 are presented in Table S1.

**Table 2:** Annual percentage changes (APCs) and their 95% confidence intervals (CIs) in the number and in the age-standardized rate of new children and new orphans influenced by paternal cancer diagnosis (adjusted and unadjusted for father’s age) in Finland during 1970–2022.

Cancer type (ICD-10)	Number of new children	Rate of new children, unadjusted for father’s age	Rate of new children, adjusted for father’s age	Number of new orphans	Rate of new orphans, unadjusted for father’s age	Rate of new orphans, adjusted for father’s age
	APC (95% CI)	APC (95% CI)	APC (95% CI)	APC (95% CI)	APC (95% CI)	APC (95% CI)
All sites together (C00-43, C45-C50, C59-96, D09.0-1, D32-33, D41-43, D45-47, D76)	0.5 (0.4, 0.6)	1.1 (1.0, 1.1)	0.7 (0.6, 0.7)	−1.6 (−1.7, −1.6)	−1.1 (−1.2, −1.0)	−1.7 (−1.8, −1.7)

By cancer type, hematological malignancies were the most common reason for a child to be newly affected by paternal cancer (Table S2, Figure S1a). During 2017–2021, the age-standardized rate of new children whose father was diagnosed with hematological malignancies increased steadily between 1970 and 2022 (Table S3).

The age-standardized prevalence of children whose father had ever been diagnosed with cancer (prevalent children) was 1,151 per 100,000 in 2022 (Table 1). The age-standardized prevalence of children whose father had been diagnosed with cancer increased from 644 per 100,000 in 1970–1974 to 1,159/100,000 in 2017 (Table 1, Figure 1d).

By cancer type, hematological malignancies accounted for the largest proportion of children ever affected by paternal cancer. Unlike most other cancer types, for which the age-standardized rate of prevalent children increased or remained stable or increased over calendar time, the rate of children whose father had lung and tracheal cancer decreased substantially (Table S2).

### Paternal orphans due to cancer deaths

The estimated age-standardized rate of orphans due to newly occurred paternal cancer death (new paternal orphans) in 2022 was 32 per 100,000 children (Table 1). A notable decrease in the average age-standardized rate of new paternal orphans was observed over time, 56 per 100,000 in 1970–1974 to 36 per 100,000 in 2017–2021, corresponding to an estimated annual decrease of 1.1% between 1970 and 2022 (Tables 1 and 2, Figure 1c). After adjusting for fathers’ age, the age-standardized rate of new paternal orphans decreased by 1.7% per year between 1970 and 2022 (Table 2, Figure 1c). Changepoints in the age-standardized rate between 1970 and 2022 are presented in Table S4.

Over a prolonged period, lung and tracheal cancers were the most common cancers associated with paternal loss in children, whereas in the most recent period, CNS malignancies became an equally common cause (Table S2, Figure S2a). A decrease in the age-standardized rate of new paternal orphans over time was observed for almost all cancer types (Table S3, Figure S2a).

In 2022, the estimated age-standardized prevalence orphans due to paternal cancer death (prevalent paternal orphans) was 228 per 100,000 children (Table 1). The prevalence of paternal orphans has reduced by almost half from 396 per 100,000 in 1970–1974 to 236 per 100,000 in 2017–2021 (Table 1, Figure 1d).

By cancer type, the age-standardized prevalence of paternal orphans over time was highest for CNS malignancies, lung and tracheal cancer and hematological malignancies (Table S2). The age-standardized prevalence of paternal orphans decreased for most cancer types from 1970–1974 to 2017–2021(Table S2, Figure S2b).

## Discussion and conclusion

Using a model-based estimation approach, this study estimated the age-standardized rates and number of children affected by paternal cancer and paternal orphanhood, and assessed trends in 1970–2022 and variation by cancer type. Over time, the age-standardized rate of children affected by paternal cancer increased, while paternal orphanhood declined.

We estimated that, in 2022, 1,832 new children were affected by a paternal cancer diagnosis. Overall, 17,987 men were diagnosed with cancer in 2022 [9], indicating that an estimated 10.2% had children younger than 18 years of age. The increase in both the age-standardized rate of new and prevalent children with paternal cancer aligns with the increasing overall cancer incidence in the male population [9].

During 1990–2022, the annual number of children born to Finnish fathers decreased from 65,549 to 44,764 with a steeper decline since 2010 [17]. On 31st of December 2022, there were 1,026,192 children aged 0–17 years living in Finland [12]. In 2022, we estimated a total of 12,704 children whose father had been diagnosed with cancer during the child’s lifetime, accounting for approximately 1.2% of the entire child population. The average annual number of children whose father had been diagnosed with cancer during a child’s lifetime was 9,228 in 1970–1974 and 8,488 in 1993–1997. During 1972–1974, there were on average 1,301,063 children under the age of 18 living in Finland annually, compared to 1,164,707 in 1993–1997 [12]. This suggests that approximately 0.7% of children in both 1970–1974 and 1993–1997 had a father who had been diagnosed with cancer. These estimates are somewhat lower than those reported a Finnish Birth Cohort study, where 1.8% of children under 18 years of age had a father with cancer [6]. A Danish study found that 2.1% of children under 18 years of age, born between 1978 and 1984, had a father with cancer [7] and a Swedish study identified an even higher proportion, 3.4%, among children born between 1961 and 2003 [8]. The proportions observed in these studies are higher than in our study, however, our estimates are based on aggregated fertility data, whereas the Danish and Swedish studies relied on individually linked register data. In addition, cancer incidence among men is higher in Denmark than in the other Nordic Countries [20], which may partly explain the differences. In Sweden, cancer incidence has been similar than in Finland since the 1980s, although slightly lower in earlier decades. Furthermore, missing data on fertility before 1990 in our study may have affected the estimates to some extent. Male fertility also varies accross the Nordic countries, with childlessness being more common among Finnish men than among their counterparts in the other Nordic countries, as shown in a birth cohort study of men born in the 1960s [21]. However, cohort total fertility has been lowest in Denmark and highest in Norway, with no subtansial differences between the Nordic countries.

Hematological malignancies were the most common cancer type among both new and prevalent cases of children affected by paternal cancer, likely reflecting the younger age distribution of patients with hematological cancers [10]. The age-standardized rate of children affected by paternal lung and tracheal cancer was decreased considerably for both new and prevalent cases, likely reflecting changes in daily smoking prevalence, which declined from 58% to 28% from 1960 to 2000 in Finland. Among working-age individuals (aged 20–64 years), daily smoking declined further from 28% in 1996 to 12% in 2022 [22]. Given the long latency period of lung and tracheal cancer, often spanning several decades [23], these long-term reductions in smoking are consistent with the observed declines in cancer incidence and mortality.

The decrease in the age-standardized rate of new paternal orphans, leading to a decrease in the rate of prevalent orphans, reflects the overall decline in cancer mortality among the male population [9]. While maternal orphanhood due to cancer has been estimated both globally and regionally [2, 24], there remains a gap in knowledge regarding the number of paternal orphans resulting from cancer. Most likely, the lack of reliable data on male fertility has limited the feasibility of such estimates. In this study, we were able to use accurate paternal fertility data, enabling us to produce reliable estimates using a model-based approach, at least for the most recent decades.

Our estimates indicate that lung and tracheal cancer were the leading causes of paternal orphanhood until recently, when CNS malignancies reached similar levels for new paternal orphanhood and surpassed lung and tracheal cancer as the most common cause of prevalent orphanhood. These patterns align with the leading causes of cancer mortality and years of life lost among Finnish men [9].

The average age at fatherhood has increased during our study period, from 28.3 years in 1985 to 32.2 years in 2023 [12]. This is reflected in the fathers’ age-adjusted model for the APC in the age-standardized rate of new children affected by paternal cancer and paternal orphans due to cancer. When adjusting for father’s age, the increase in the rate of new children became less evident, whereas the decrease in the rate of new orphans was more pronounced. The median age of the child at the time of paternal cancer diagnosis decreased slightly, most likely also reflecting the increase in the average age at fatherhood.

The impact of paternal cancer or paternal death likely depends on the father–child relationship. In 2022, 72% of Finnish children lived with both parents and 28% with one parent [25]. Although these statistics do not account for joint residency, studies indicate that about one-third of children with separated parents alternate between households [26]. Thus, most children in Finland live with their father at least part‑time, suggesting that paternal cancer or the loss of a father can affect a large proportion of children.

### Strengths and limitations

This study had some limitations. The results on the number and rates of children and orphans are based on model-derived estimates using register-based fertility data from Statistics Finland and do not present individual-level data on parenthood and family relationships.

In particular, the lack of individual-level fertility data among men diagnosed with or dying from cancer represents a key limitation. Our estimates assume that the age-specific number of children among men affected by cancer is similar to that in the general male population in Finland, which may introduce bias. This is especially relevant if cancer risk factors are associated with fertility.

We did not account for the potential effects of cancer type on fertility in our analysis. Although some studies have shown that cancer type generally does not substantially affect future fertility [27], differences in cancer type and treatment may still influence the likelihood of having children. Liable paternal fertility data were available from 1990 onward meaning that the estimates for the number of orphans and number of children whose father was diagnosed with cancer are most reliable from the year 2007 onward. A future aim is to use individual-level data to strengthen the findings and to explore underlying factors that may affect children influenced by paternal cancer diagnosis and death.

The main strength of the study is the use of validated, nationwide population-based cancer registry data [16], along with high-quality statistical data on births [17] and causes of death [11] collected by Statistics Finland. The availability of reliable paternal fertility data is uncommon and highlights the novelty of this study. The statistical approach to estimate the number of children and orphans has previously been presented and assessed by the International Agency for Research on Cancer (IARC) using maternal cancer and fertility data [2].

## Conclusions

Cancer affects many families, and children are in a particularly vulnerable position when a parent is diagnosed with cancer. Fortunately, fewer children are experiencing paternal cancer death; however, simultaneously, more children are affected by paternal cancer diagnosis. Fathers are also developing and dying from different types of cancer than in the past. Serious parental illness can have significant consequences for a child’s well-being, and affected children should be offered support tailored to their specific needs. Future research should aim to identify and address the challenges faced by children affected by parental cancer to enable more targeted interventions, including social and financial support as well as accessible mental health services.

## Supplementary Material



## Data Availability

Cancer Registry data are publicly available at the Finnish Cancer Registry Statistic Application: https://tilastot.syoparekisteri.fi/syovat/. Data on paternal fertility rates are also publicly available at the StatFin database: https://pxdata.stat.fi/PxWeb/pxweb/en/StatFin/.
