# Supplementary material for: Estimated number of children affected by paternal cancer diagnosis and death in Finland during 1970–2022: a population-based registry study
Source: Acta Oncol. 2026 Jul 30;65:45653. doi: 10.2340/1651-226X.2026.45653 (PMC13430636; doi:10.2340/1651-226X.2026.45653)

Supplementary material has been published as submitted. It has not been copyedited, or typeset by Acta Oncologica

Supplementary Material

Estimated number of children affected by paternal cancer diagnosis and death in Finland during 1970–2022: a population-based registry study

Authors: Anniina Kyrönlahti, Eetu Mäkinen, Karri Seppä, Tea Lallukka, Janne Pitkäniemi

Table of Contents

Statistical methods 2
References 3
Table S1 4
Table S2 6
Table S3 8
Table S4 10
Figure S1 12
Figure S2 12

Statistical methods

For the estimation of the number of orphans we followed the methodology introduced by Guida et al in 2022 (1) and further applied it to estimate the number of children whose father was diagnosed with cancer and the corresponding age-standardized rates. The estimates were calculated from 1970 to 2022.

The data included mortality rates $M_{y,ca}$, by calendar year y $(1953, \dots, 2022)$ and child's age ca $(0, \dots, 17)$, and numbers of cancer deaths $D_{y,fa,t}$, by calendar year y $(1953, \dots, 2022)$, father's age fa $(15, \dots, 100)$ and cancer type t from Statistics Finland, and numbers of cancer cases $C_{y,fa,t}$, by calendar year y $(1953, \dots, 2022)$, father's age fa $(15, \dots, 100)$ and cancer type t from the Finnish Cancer Registry. The mortality rates used were the year and age-wise averages of the mortality rates of boys and girls.

Number of births by father's age were observed for calendar years y $(1990, \dots, 2022)$ and father's ages fa $(15, \dots, 59)$ from Statistics Finland (2). Number of births for fathers aged 60 and over were observed in one category, and their distribution in one-year groups of age was estimated with an age-period Poisson model. Additionally, fathers' ages in births where father's age was unknown were assumed to be distributed similarly as in the observed births and allocated based on the yearly age distribution.

Total number of births were observed from 1953 to 1989. Number of births by father's age for 1953-1989 were estimated with an age-period-cohort Poisson model and scaled so that the yearly sums of births by father's age equalled the observed yearly total number of births. Numbers of births were then converted to fertility rates $FR_{y,fa}$ by scaling with population sizes from Statistics Finland (3).

The probability that a child born in year yob is alive in year y when their father has not died of cancer is denoted by $P(alive_y | yob)$ and was estimated based on the mortality rates by $exp\left\{-\sum_{i=yob}^{y-1} M_{i,i-yob}\right\}$.

We denote the probability that a child born in year yob is alive in year y, given that their father died of cancer in year fd by $P(alive_y | yob, fd)$ which was estimated by $exp\left\{-\sum_{i=yob}^{fd-1} M_{i,i-yob} - 1.25 \cdot \sum_{i=fd}^{y-1} M_{i,i-yob}\right\}$. The coefficient 1.25 is the mortality HR for children who have lost a parent (1).

For cases of new orphans and new children with paternal cancer we estimated the **A**verage number of living **C**hildren by father's age fa $(15, \dots, 100)$ and calendar year y $(1970, \dots, 2022)$ as

$$AC_{y,fa} = \sum_{i=y-17}^{y} FR_{i,fa-(y-i)} \cdot P(alive_y | i).$$

Meanwhile, in the case of total orphans, we instead calculated the **A**verage number of living **O**rphans (**P**revalent) by calendar years y $(1970, \dots, 2022)$ given that the father has died of cancer in year fd as

$$AOP_{y,fa} = \sum_{fd=y-17}^{y} \sum_{i=y-17}^{fd} FR_{i,fa-(y-i)} \cdot P(alive_y | i, fd)$$

and in the case of total children with father diagnosed ever during child's lifetime we estimate the **A**verage number of **C**hildren (**P**revalent) by calendar year y $(1970, \dots, 2022)$ as

$$ACP_{y,fa} = \sum_{fd=y-17}^{y} \sum_{i=y-17}^{fd} FR_{i,fa-(y-i)} \cdot P(alive_y | i).$$

Number of **N**ew children whose father was **D**iagnosed with cancer type t by year y $(1970, \dots, 2022)$:

$$ND_{y,t} = \sum_{fa=15}^{100} C_{y,fa,t} \cdot AC_{y,fa}.$$

Number of **N**ew **O**rphans due to cancer type t by year y $(1970, \ldots, 2022)$:

$$NO_{y,t} = \sum_{fa=15}^{100} D_{y,fa,t} \cdot AC_{y,fa}.$$

Number of **T**otal children whose father has been **D**iagnosed with cancer type t during child's lifetime by year y $(1970, \ldots, 2022)$:

$$TD_{y,t} = \sum_{fa=15}^{100} C_{y,fa,t} \cdot ACP_{y,fa}.$$

Number of **T**otal **O**rphans due to cancer type t by year y $(1970, \ldots, 2022)$:

$$TO_{y,t} = \sum_{fa=15}^{100} D_{y,fa,t} \cdot AOP_{y,fa}.$$

The age-standardized rates for paternal orphanhood due to cancer,

$$ASR_y = 100.000 \cdot \sum_{a=0}^{17} w_a \cdot \frac{NOA_{a,y}}{pop_{a,y}},$$

where w_a is the weight for the 1-year age group a, as calculated from the World (WHO 2000–2025) Standard population, $pop_{a,y}$ is the mean population of age group a in year y, and $NOA_{a,y}$ is the age-specific number of new orphans due to cancer in year y:

$$NOA_{a,y} = \sum_{t} \sum_{fa=15+a}^{100-(17-a)} C_{y,fa,t} \cdot FR_{y-a,fa-a} \cdot P\left(alive_y \middle| y-a\right).$$

References

1. Guida F, Kidman R, Ferlay J, Schüz J, Soerjomataram I, Kithaka B, et al. Global and regional estimates of orphans attributed to maternal cancer mortality in 2020. Nat Med. 2022 Dec;28(12):2563–72.
2. Official Statistics of Finland (OSF). Births [online publication]. Helsinki: Statistics Finland; [cited 2025 Feb 12]. Available from: https://stat.fi/en/statistics/synt
3. Official Statistics of Finland (OSF). Population structure [online publication]. Helsinki: Statistics Finland; [cited 2025 Feb 25]. Available from: https://stat.fi/en/statistics/vaerak

Table S1. Changepoint sections and their 95% confidence intervals (CIs) of the annual percentage changes (APCs) in the number and in the age-standardized rate of new children influenced by paternal cancer diagnosis (adjusted and unadjusted for father's age) in Finland during 1970–2022 by cancer type (ICD-10 coding based categorization).

Cancer type (ICD-10)	Number of new children		Rate of new children, unadjusted for father's age		Rate of new children, adjusted for father's age	
	Changepoint section	Section APC (95% CI)	Changepoint section	Section APC (95% CI)	Changepoint section	Section APC (95% CI)
All sites together (C00-43,C45-C50,C59-96,D09.0-1,D32-33,D41-43,D45-47,D76)	2016-2022	0.4 (-0.5, 1.3)	2016-2022	0.5 (-0.4, 1.4)	1993-2022	1.2 (1.1, 1.3)
	1987-2016	1.7 (1.5, 1.8)	1991-2016	2.0 (1.9, 2.2)	1970-1993	0.0 (-0.2, 0.2)
	1970-1987	-1.3 (-1.5, -1.0)	1970-1991	0.0 (-0.2, 0.2)		
Bladder and urinary tract (C65-68,D09.0-1,D41.1-9)	1970-2022	-	1970-2022	-	1970-2022	-
Brain, meninges and CNS* (C70-72,D32-33,D42-43)	2018-2022	7.6 (0.3, 15.5)	2013-2022	3.6 (1.8, 5.6)	2012-2022	3.8 (1.9, 5.7)
	1970-2018	0.8 (0.6, 1.0)	1985-2013	0.6 (0.2, 1.1)	1985-2012	0.3 (-0.1, 0.8)
			1970-1985	2.9 (1.7, 4.0)	1970-1985	3.0 (1.9, 4.2)
Colon and rectum (C18-20)	1985-2022	2.1 (1.8, 2.3)	1970-2022	-	1970-2022	-
	1970-1985	-0.2 (-1.4, 1.0)				
Gallbladder, bile ducts (C23-24)	2011-2022	6.5 (1.1, 12.1)	1970-2022	-	1970-2022	-
	1970-2011	-0.3 (-1.2, 0.7)				
Hematological (C81-96,D45-47,D76)	1980-2022	1.1 (1.0, 1.3)	1970-2022	-	1970-2022	-
	1970-1980	-0.8 (-2.3, 0.8)				
Kidney (C64)	1970-2022	-	1970-2022	-	1970-2022	-
Larynx, epiglottis (C32)	1977-2022	-2.2 (-2.7, -1.6)	1977-2022	-1.8 (-2.4, -1.2)	1973-2022	-2.9 (-3.4, -2.4)
	1970-1977	-12.0 (-16.3, -7.5)	1970-1977	-10.0 (-15.1, -4.7)	1970-1973	-17.5 (-27.4, -6.3)
Liver (C22)	1970-2022	-	1970-2022	-	1970-2022	-
Lung, trachea (C33-34)	1986-2022	-1.7 (-2.0, -1.4)	1988-2022	-1.4 (-1.8, -1.1)	1970-2022	-
	1980-1986	-7.2 (-9.7, -4.7)	1980-1988	-5.2 (-7.0, -3.4)		
	1970-1980	-3.0 (-4.2, -1.7)	1970-1980	-1.8 (-3.0, -0.5)		
Melanoma of the skin (C43)	2001-2022	3.4 (2.8, 4.0)	1995-2022	3.4 (3.0, 3.9)	1997-2022	3.3 (2.9, 3.8)
	1986-2001	0.3 (-0.8, 1.5)	1987-1995	-2.0 (-4.5, 0.5)	1987-1997	-1.8 (-3.9, 0.4)
	1970-1986	3.5 (2.4, 4.7)	1970-1987	5.0 (3.8, 6.2)	1970-1987	5.0 (3.8, 6.2)

Mouth, pharynx (C00-14)	1987-2022	1.9 (1.5, 2.3)	1987-2022	2.2 (1.8, 2.6)	1987-2022	1.5 (1.1, 2.0)
	1970-1987	-2.8 (-4.1, -1.5)	1970-1987	-1.6 (-2.8, -0.4)	1970-1987	-1.9 (-3.0, -0.7)
Oesophagus (C15)	1970-2022	-	1970-2022	-	1970-2022	-
Pancreas (C25)	1975-2022	0.6 (0.3, 0.9)	1974-2022	1.0 (0.6, 1.3)	1973-2022	0.0 (-0.3, 0.4)
	1970-1975	-12.1 (-19.4, -4.2)	1970-1974	-10.7 (-18.1, -2.6)	1970-1973	-12.8 (-22.6, -1.8)
Prostate (C61)	2004-2022	-0.9 (-1.4, -0.3)	2004-2022	-0.5 (-1.0, 0.1)	2004-2022	-1.7 (-2.2, -1.1)
	1991-2004	19.8 (17.9, 21.8)	1992-2004	20.1 (18.1, 22.1)	1992-2004	16.8 (14.9, 18.8)
	1970-1991	1.4 (-0.1, 2.9)	1970-1992	2.6 (1.1, 4.1)	1970-1992	2.3 (0.8, 3.9)
Soft tissues (C48-49)	1970-2022	-	1970-2022	-	1970-2022	-
Stomach (C16)	1988-2022	-2.2 (-2.6, -1.7)	1970-2022	-	1970-2022	-
	1970-1988	-4.1 (-4.9, -3.2)				
Testis (C62)	2015-2022	-3.9 (-7.3, -0.5)	2015-2022	-3.0 (-5.7, -0.2)	2015-2022	-2.6 (-5.3, 0.2)
	1994-2015	5.3 (4.5, 6.2)	1995-2015	5.8 (4.8, 6.8)	1995-2015	6.2 (5.3, 7.1)
	1970-1994	1.4 (0.5, 2.3)	1970-1995	2.0 (1.2, 2.9)	1970-1995	2.4 (1.5, 3.3)
Thyroid gland (C73)	1970-2022	-	1972-2022	2.4 (2.1, 2.8)	1972-2022	2.3 (2.0, 2.7)
			1970-1972	69.2 (15.3, 148.3)	1970-1972	82.3 (22.0, 172.3)

*Central nervous system

Table S2. Estimated number and age-standardized rate of children under 18 years of age influenced by paternal cancer diagnosis (at the age of ≥15 years) and paternal cancer death in Finland by follow-up periods and cancer type (ICD-10 coding based categorization).

		New children whose father was diagnosed with cancer		Children whose father was ever diagnosed with cancer		New orphans		Prevalent orphans			
Cancer type (ICD-10)	Period	Average annual number	Average annual rate	Average annual number	Average annual rate	Average annual number of new orphans	Average annual rate of new orphans	Average number of prevalent orphans	Average rate of prevalent orphans	Median child age at father's cancer diagnosis	Median child age at father's cancer death
Bladder and urinary tract (C65-68,D09.0-1,D41.1-9)	2017-2021	64	5.9	411	37.5	5	0.5	34	3.1	12.6	13.6
	1993-1997	55	4.7	350	29.6	6	0.5	32	2.7	12.4	13.2
	1970-1974	47	3.4	224	15.7	12	0.8	51	3.5	12.8	13.6
Brain, meninges and CNS* (C70-72,D32-33,D42-43)	2017-2021	142	13.3	1077	98.6	45	4.2	380	34.7	10.4	11.2
	1993-1997	118	10.1	945	80.1	52	4.4	416	35.2	10.1	10.4
	1970-1974	86	6.4	740	52.3	59	4.3	553	38.9	10.3	10.7
Colon and rectum (C18-20)	2017-2021	188	17.4	1131	103.3	47	4.3	275	25.2	12.0	12.3
	1993-1997	115	9.8	704	59.5	41	3.5	257	21.7	11.9	12.3
	1970-1974	91	6.5	654	45.8	48	3.4	327	22.9	12.4	12.5
Gallbladder, bile ducts (C23-24)	2017-2021	12	1.1	63	5.8	8	0.8	39	3.6	12.4	12.7
	1993-1997	7	0.6	51	4.3	5	0.5	41	3.4	12.5	12.8
	1970-1974	8	0.6	42	3.0	8	0.5	37	2.6	11.9	12.2
Hematological (C81-96,D45-47,D76)	2017-2021	275	25.7	2033	186.0	33	3.0	283	25.8	10.8	12.2
	1993-1997	225	19.2	1618	137.0	69	5.8	584	49.3	10.8	11.5
	1970-1974	187	13.7	1467	103.7	111	8.2	1010	71.2	10.8	10.6
Kidney (C64)	2017-2021	91	8.4	565	51.6	12	1.1	88	8.0	12.0	13.1
	1993-1997	75	6.3	423	35.7	23	2.0	134	11.3	12.5	12.9
	1970-1974	52	3.7	281	19.6	24	1.7	145	10.0	12.9	13.3
Larynx, epiglottis (C32)	2017-2021	9	0.8	62	5.7	2	0.2	13	1.1	13.1	13.4
	1993-1997	17	1.5	110	9.2	5	0.4	25	2.1	12.8	12.8
	1970-1974	46	3.2	345	23.7	13	0.9	90	6.2	13.5	14.0
Liver (C22)	2017-2021	26	2.4	146	13.3	16	1.5	99	9.0	12.5	12.8
	1993-1997	15	1.3	88	7.4	12	1.0	72	6.1	12.3	12.3
	1970-1974	12	0.9	56	3.9	11	0.8	51	3.6	13.2	13.3
Lung, trachea (C33-34)	2017-2021	79	7.3	530	48.4	55	5.1	353	32.2	13.2	13.4

	1993-1997	132	11.1	796	67.0	99	8.4	588	49.5	13.2	13.2
	1970-1974	305	21.2	1739	119.7	225	15.7	1315	90.5	13.8	13.9
Melanoma of the skin (C43)	2017-2021	179	16.7	1314	120.1	13	1.2	101	9.3	11.1	11.9
	1993-1997	97	8.3	770	65.1	21	1.7	167	14.1	11.0	11.9
	1970-1974	62	4.5	391	27.8	29	2.1	191	13.5	10.8	11.0
Mouth, pharynx (C00-14)	2017-2021	80	7.4	526	48.0	12	1.1	92	8.4	12.1	12.6
	1993-1997	55	4.6	323	27.3	15	1.2	90	7.6	12.3	12.2
	1970-1974	66	4.7	533	36.9	12	0.9	105	7.3	13.0	13.4
Oesophagus (C15)	2017-2021	26	2.4	137	12.5	20	1.8	95	8.7	12.9	12.8
	1993-1997	16	1.4	82	7.0	13	1.1	64	5.4	13.1	13.1
	1970-1974	12	0.8	77	5.3	9	0.6	58	4.0	14.0	14.0
Pancreas (C25)	2017-2021	48	4.4	284	25.9	33	3.1	191	17.4	13.0	13.4
	1993-1997	39	3.3	230	19.4	32	2.7	189	15.9	12.8	13.1
	1970-1974	52	3.7	283	19.7	44	3.1	253	17.6	13.0	13.1
Prostate (C61)	2017-2021	223	20.4	1210	110.3	10	0.9	50	4.6	13.9	13.9
	1993-1997	49	4.1	160	13.5	11	0.9	42	3.5	14.1	13.9
	1970-1974	17	1.2	73	5.0	6	0.4	27	1.9	14.3	14.3
Soft tissues (C48-49)	2017-2021	25	2.3	199	18.2	7	0.7	56	5.2	10.2	9.6
	1993-1997	17	1.5	160	13.6	7	0.6	65	5.5	10.1	10.3
	1970-1974	18	1.4	189	13.3	14	1.0	96	6.8	10.7	11.2
Stomach (C16)	2017-2021	36	3.3	256	23.4	19	1.7	159	14.5	12.2	12.6
	1993-1997	61	5.1	445	37.5	40	3.4	285	24.0	12.2	12.3
	1970-1974	131	9.3	1034	71.5	103	7.3	807	55.8	13.0	13.0
Testis (C62)	2017-2021	113	10.8	1232	113.1	5	0.5	43	3.9	7.0	7.5
	1993-1997	47	4.1	446	37.9	3	0.2	40	3.4	6.7	8.2
	1970-1974	36	2.8	265	19.1	9	0.7	87	6.2	8.0	7.4
Thyroid gland (C73)	2017-2021	52	4.9	408	37.4	2	0.1	9	0.9	10.1	13.1
	1993-1997	36	3.1	246	20.9	1	0.1	10	0.8	10.0	12.1
	1970-1974	20	1.5	120	8.6	4	0.3	24	1.7	9.5	11.5
Other (C17,C21,C26,C30-31,C37-41,C45-47,C50,C60,C63,C69,C74-80)	2017-2021	87	8.1	623	56.9	30	2.8	220	20.1	11.7	12.2
	1993-1997	74	6.3	538	45.5	40	3.4	268	22.6	11.5	12.1
	1970-1974	90	6.5	715	50.1	52	3.7	452	31.5	11.9	12.0

*Central nervous system

Table S3. Annual percentage changes (APCs) and their 95% confidence intervals (CIs) in the number and in the age-standardized rate of new children and new orphans influenced by paternal cancer diagnosis (adjusted and unadjusted for father's age) in Finland during 1970–2022 by cancer type (ICD-10 coding based categorization).

Cancer type (ICD-10)	Number of new children	Rate of new children, unadjusted for father's age	Rate of new children, adjusted for father's age	Number of new orphans	Rate of new orphans, unadjusted for father's age	Rate of new orphans, adjusted for father's age
	APC (95% CI)	APC (95% CI)	APC (95% CI)	APC (95% CI)	APC (95% CI)	APC (95% CI)
Bladder and urinary tract (C65-68,D09.0-1,D41.1-9)	0.7 (0.4, 0.9)	1.1 (0.9, 1.3)	0.3 (0.1, 0.6)	-1.7 (-2.4, -0.9)	-1.0 (-1.7, -0.4)	-1.7 (-2.4, -1.0)
Brain, meninges and CNS* (C70-72,D32-33,D42-43)	1.2 (1.0, 1.5)	1.8 (1.5, 2.1)	1.8 (1.5, 2.0)	-0.5 (-0.7, -0.2)	0.0 (-0.3, 0.2)	-0.3 (-0.5, 0.0)
Colon and rectum (C18-20)	1.4 (1.2, 1.6)	2.1 (2.0, 2.3)	1.5 (1.3, 1.7)	-0.3 (-0.6, 0.1)	0.4 (0.2, 0.7)	-0.3 (-0.5, 0.0)
Gallbladder, bile ducts (C23-24)	1.1 (0.4, 1.8)	1.1 (0.4, 1.7)	0.4 (-0.3, 1.0)	-0.2 (-0.9, 0.5)	0.3 (-0.4, 1.0)	-0.4 (-1.1, 0.4)
Hematological (C81-96,D45-47,D76)	0.8 (0.6, 0.9)	1.4 (1.2, 1.5)	1.1 (1.0, 1.2)	-2.4 (-2.7, -2.2)	-2.1 (-2.4, -1.9)	-2.4 (-2.6, -2.1)
Kidney (C64)	1.2 (0.9, 1.4)	1.6 (1.4, 1.8)	1.0 (0.8, 1.2)	-1.2 (-1.6, -0.8)	-0.9 (-1.4, -0.4)	-1.4 (-1.9, -0.9)
Larynx, epiglottis (C32)	-3.6 (-4.0, -3.1)	-3.0 (-3.4, -2.5)	-3.9 (-4.4, -3.4)	-3.5 (-4.4, -2.7)	-3.1 (-3.9, -2.2)	-3.8 (-4.7, -3.0)
Liver (C22)	1.8 (1.4, 2.3)	2.3 (1.8, 2.8)	1.6 (1.2, 2.1)	1.2 (0.7, 1.7)	1.7 (1.1, 2.2)	0.9 (0.4, 1.5)
Lung, trachea (C33-34)	-2.6 (-2.8, -2.5)	-2.1 (-2.3, -1.9)	-3.3 (-3.4, -3.2)	-2.9 (-3.1, -2.7)	-2.3 (-2.5, -2.1)	-3.4 (-3.6, -3.3)
Melanoma of the skin (C43)	2.5 (2.3, 2.8)	3.1 (2.8, 3.3)	2.9 (2.6, 3.1)	-1.7 (-2.1, -1.3)	-1.2 (-1.6, -0.8)	-1.5 (-1.9, -1.1)
Mouth, pharynx (C00-14)	0.4 (0.1, 0.7)	0.9 (0.7, 1.2)	0.4 (0.2, 0.7)	0.3 (-0.2, 0.8)	0.7 (0.3, 1.2)	0.2 (-0.2, 0.7)
Oesophagus (C15)	1.9 (1.5, 2.4)	2.3 (1.9, 2.8)	1.6 (1.1, 2.1)	1.7 (1.2, 2.2)	2.1 (1.6, 2.7)	1.4 (0.8, 1.9)
Pancreas (C25)	-0.7 (-1.1, -0.3)	-0.1 (-0.5, 0.3)	-0.9 (-1.3, -0.4)	-1.2 (-1.6, -0.7)	-0.6 (-1.0, -0.1)	-0.9 (-1.2, -0.6)

Prostate (C61)	4.9 (4.5, 5.3)	5.5 (5.1, 5.9)	4.2 (3.8, 4.6)	0.9 (0.2, 1.6)	1.5 (0.8, 2.3)	0.1 (-0.7, 0.9)
Soft tissues (C48-49)	0.4 (0.1, 0.8)	0.9 (0.5, 1.3)	0.8 (0.4, 1.2)	-1.8 (-2.6, -1.1)	-1.0 (-1.6, -0.4)	-0.8 (-1.5, -0.2)
Stomach (C16)	-2.8 (-3.1, -2.6)	-2.4 (-2.6, -2.1)	-2.9 (-3.1, -2.7)	-3.3 (-3.6, -3.0)	-2.8 (-3.1, -2.6)	-3.4 (-3.7, -3.1)
Testis (C62)	2.2 (1.8, 2.6)	2.7 (2.3, 3.0)	3.1 (2.8, 3.5)	-1.1 (-1.9, -0.2)	-0.7 (-1.6, 0.1)	-0.5 (-1.3, 0.4)
Thyroid gland (C73)	2.2 (1.8, 2.5)	4.4 (3.2, 5.7)	4.6 (3.3, 6.0)	-1.4 (-2.7, -0.2)	-1.3 (-2.6, 0.0)	-1.7 (-3.0, -0.3)

*Central nervous system

Table S4. Changepoint sections and their 95% confidence intervals (CIs) of the annual percentage changes (APCs) in the number and in the rate of new orphans influenced by paternal cancer diagnosis (adjusted and unadjusted for father's age) in Finland during 1970–2022 (unadjusted and adjusted for father's age) by cancer type (ICD-10 coding based categorization).

Cancer type	Number of new orphans		Rate of new orphans, unadjusted for father's age		Rate of new orphans, adjusted for father's age	
	Changepoint section	Section APC (95% CI)	Changepoint section	Section APC (95% CI)	Changepoint section	Section APC (95% CI)
All sites together (C00-43,C45-C50,C59-96,D09.0-1,D32-33,D41-43,D45-47,D76)	1993-2022	-1.2 (-1.4, -1.0)	1984-2022	-0.8 (-0.9, -0.6)	1970-2022	-
	1990-1993	3.4 (-2.9, 10.2)	1970-1984	-2.0 (-2.4, -1.5)		
	1970-1990	-3.0 (-3.3, -2.7)				
Bladder and urinary tract (C65-68,D09.0-1,D41.1-9)	1984-2022	-0.1 (-1.3, 1.0)	1970-2022	-	1970-2022	-
	1970-1984	-5.7 (-9.5, -1.7)				
Brain, meninges and CNS*(C70-72,D32-33, D42-43)	1970-2022	-	1970-2022	-	1970-2022	-
Colon and rectum (C18-20)	1983-2022	0.5 (0.1, 1.0)	1970-2022	-	1970-2022	-
	1970-1983	-2.6 (-4.6, -0.7)				
Gallbladder, bile ducts (C23-24)	1970-2022	-	1970-2022	-	1970-2022	-
Hematological (C81-96,D45-47,D76)	1970-2022	-	1998-2022	-3.0 (-3.8, -2.2)	1986-2022	-3.0 (-3.5, -2.6)
			1970-1998	-1.4 (-1.9, -0.9)	1970-1986	-0.8 (-1.9, 0.3)
Kidney (C64)	1970-2022	-	1996-2022	-2.3 (-3.6, -1.1)	1989-2022	-2.9 (-3.8, -2.1)
			1970-1996	0.6 (-0.4, 1.6)	1970-1989	1.2 (-0.4, 2.8)
Larynx, epiglottis (C32)	1970-2022	-	1970-2022	-	1970-2022	-
Liver (C22)	1970-2022	-	1970-2022	-	1970-2022	-
Lung, trachea (C33-34)	1988-2022	-2.1 (-2.4, -1.7)	1991-2022	-1.8 (-2.2, -1.4)	1970-2022	-
	1970-1988	-4.4 (-5.1, -3.8)	1970-1991	-3.1 (-3.6, -2.5)		
Melanoma of the skin (C43)	1970-2022	-	1970-2022	-	1970-2022	-
Mouth, pharynx (C00-14)	1970-2022	-	1970-2022	-	1970-2022	-
Oesophagus (C15)	1970-2022	-	1970-2022	-	1970-2022	-

Pancreas (C25)	1974-2022	0.0 (-0.3, 0.3)	1974-2022	0.4 (0.0, 0.7)	1970-2022	-
	1970-1974	-14.3 (-24.3, -3.0)	1970-1974	-11.5 (-19.5, -2.6)		
Prostate (C61)	2002-2022	-1.5 (-3.7, 0.7)	1998-2022	-0.9 (-2.7, 1.0)	1994-2022	-2.7 (-4.2, -1.2)
	1970-2002	2.4 (1.1, 3.8)	1970-1998	3.6 (2.0, 5.2)	1970-1994	3.5 (1.3, 5.7)
Soft tissues (C48-49)	1979-2022	-0.6 (-1.5, 0.3)	1970-2022	-	1970-2022	-
	1970-1979	-7.4 (-13.2, -1.2)				
Stomach (C16)	1987-2022	-2.4 (-3.0, -1.8)	1970-2022	-	1970-2022	-
	1970-1987	-5.0 (-6.0, -4.0)				
Testis (C62)	2004-2022	6.6 (1.8, 11.5)	2004-2022	6.8 (2.1, 11.8)	2001-2022	6.2 (2.5, 10.1)
	1970-2004	-4.9 (-6.3, -3.5)	1970-2004	-4.5 (-5.9, -3.1)	1970-2001	-4.8 (-6.5, -3.1)
Thyroid gland (C73)	1970-2022	-	1970-2022	-	1970-2022	-

*Central nervous system

Figure S1. Estimated annual age-standardized rate of new children (A) and prevalent children (B) under 18 years of age influenced by paternal cancer diagnosis (diagnosed at the age of ≥15 years) in Finland during 1970–2022 by cancer type (ICD-10 coding based categorization).

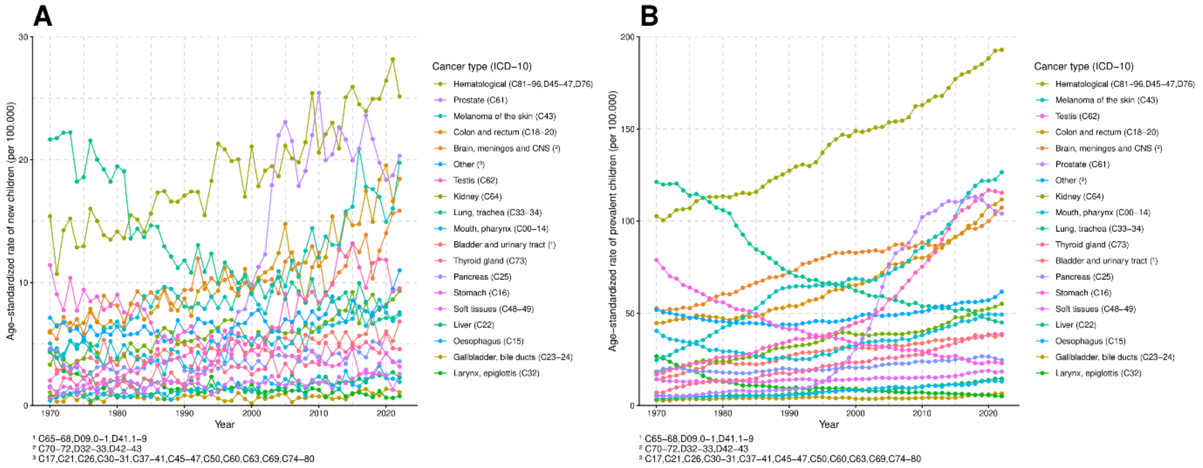

Figure S2. Estimated annual age-standardized rate of new children (A) and prevalent children (B) under 18 years of age influenced by paternal cancer death (orphans) in Finland during 1970–2022 by cancer type (ICD-10 coding based categorization).

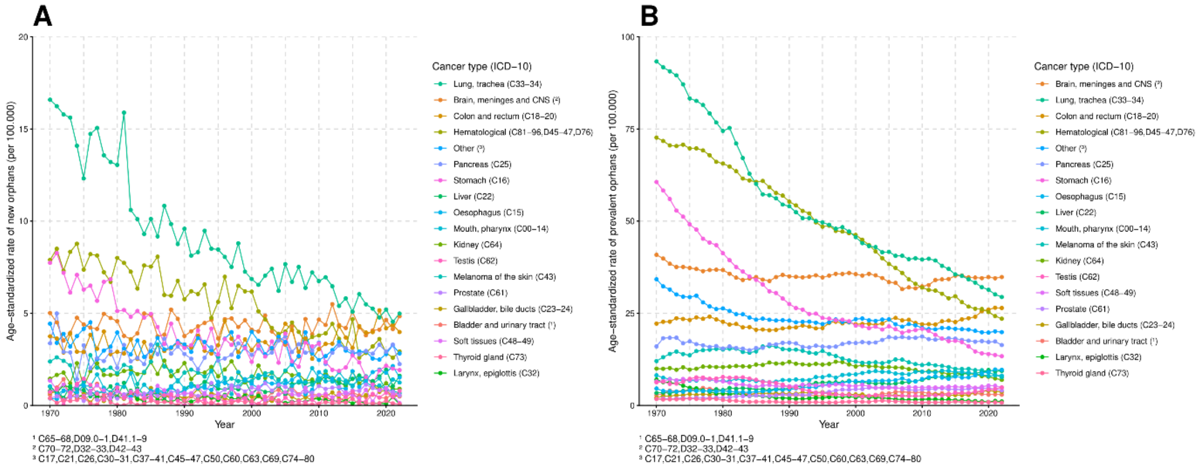

Supplement: Supplementary file 1 [file AO-65-45653-s1.pdf]
